# Estimating COVID-19 Infections, Hospitalizations, and Deaths Following the US Vaccination Campaigns During the Pandemic

**DOI:** 10.1001/jamanetworkopen.2021.42725

**Published:** 2022-01-11

**Authors:** Thomas N. Vilches, Seyed M. Moghadas, Pratha Sah, Meagan C. Fitzpatrick, Affan Shoukat, Abhishek Pandey, Alison P. Galvani

**Affiliations:** 1 Agent-Based Modelling Laboratory, York University, Toronto, Ontario, Canada; 2 Center for Infectious Disease Modeling and Analysis, Yale School of Public Health, New Haven, Connecticut; 3 Center for Vaccine Development and Global Health, University of Maryland School of Medicine, Baltimore

## Abstract

This decision analytic modeling study uses a simulation model to evaluate the association of US COVID-19 vaccination campaigns with infections, hospitalizations, and deaths.

## Introduction

The COVID-19 pandemic has caused more than 745 000 deaths in the US. However, the toll might have been higher without the rapid development and delivery of effective vaccines. As of October 28, 2021, 69% of 258 million US adults had been fully vaccinated.

Quantifying the population impact of COVID-19 vaccination can inform future vaccination strategies. Randomized clinical trials have established individual-level efficacy of authorized vaccines against the original strain, which exceeds 90% in preventing symptomatic and severe disease.^1,2,3^ However, the population-level effectiveness of the vaccination campaign in the US, in terms of association with reduced infections, hospitalizations, and deaths, is not as well documented, and we evaluated this using a simulation model.

## Methods

This decision analytic model adheres to Consolidated Health Economic Evaluation Reporting Standards (CHEERS) reporting guideline. The institutional review of this study was waived by York University for the use of publicly available, deidentified data of the COVID-19 infections, deaths, and vaccination. Informed consent was not required to access the data.

We expanded our previous agent-based model^4^ to include transmission dynamics of the Alpha (B.1.1.7), Gamma (P.1), and Delta (B.1.617.2) variants in addition to the original strain (eMethods in the Supplement). The model was parameterized with the US demographics and age-specific risks of severe COVID-19 outcomes (eTable 1 and eTable 2 in the Supplement).^5^ A 2-dose vaccination strategy was implemented based on the daily vaccines administered in different age groups.^6^ Vaccine efficacies against infection, symptomatic disease and severe disease after each dose and for each variant were derived from published estimates (eTable 3 in the Supplement). The model was calibrated and fitted to reported national level incidence from October 1, 2020, to June 30, 2021 (eMethods in the Supplement).

We simulated pandemic trajectory under 2 counterfactuals: a no vaccination scenario and a program that achieved only half the daily vaccination rate of actual rollout. For each scenario, cumulative infections, hospitalizations, and deaths were compared with the simulated trends under the US vaccination program.

Credible intervals (CrIs) were generated from simulation outputs using the bias-corrected and accelerated bootstrap method (with 500 replications) in June 2021. The model was implemented in Julia Language Programming, version 1.6 (Julia), and outputs were analyzed in MATLAB, version 2017a (MathWorks). No significance tests were performed for this simulation study.

## Results

**Figure 1.**
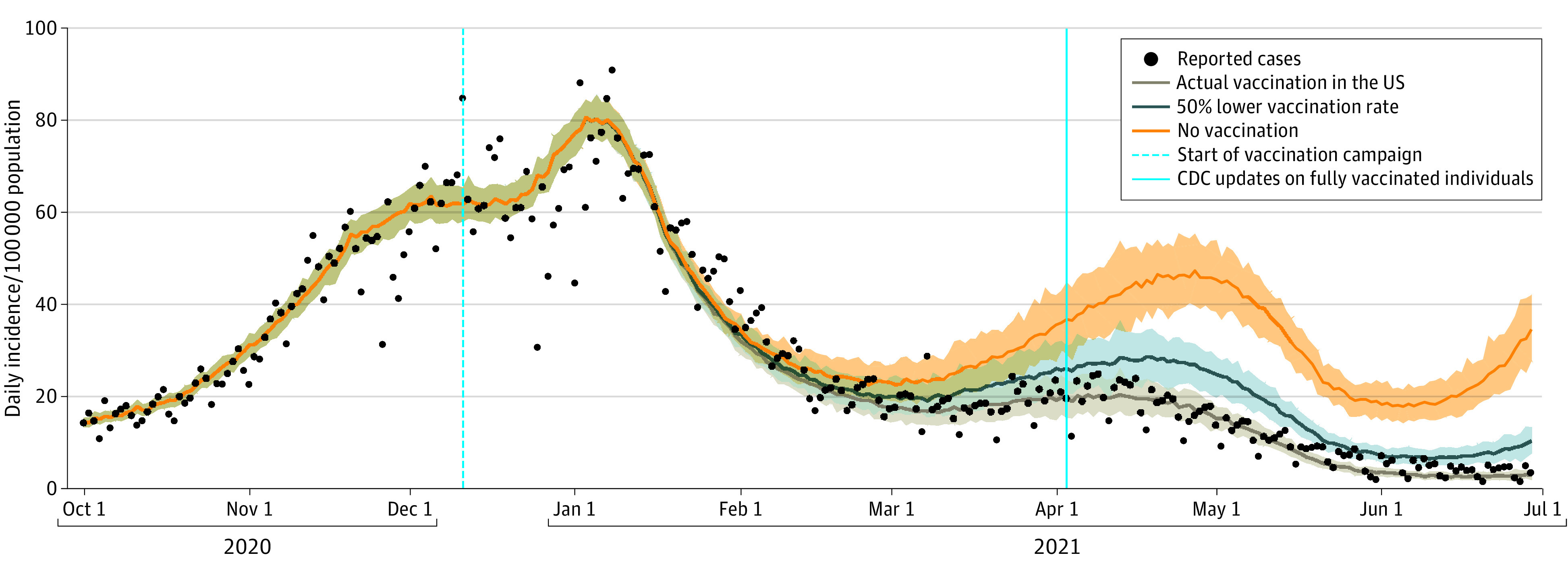
Association of COVID-19 Vaccination With Infections Projected daily incidence per 100 000 population caused by different variants of SARS-CoV-2 with actual vaccination rollout in the US, a temporal vaccination rate reduced to half of the actual pace, and without vaccination. Black dots are reported incidence per 100 000 population, and shaded areas represent the range of uncertainty (95% CrI) in the projections.

**Figure 2.**
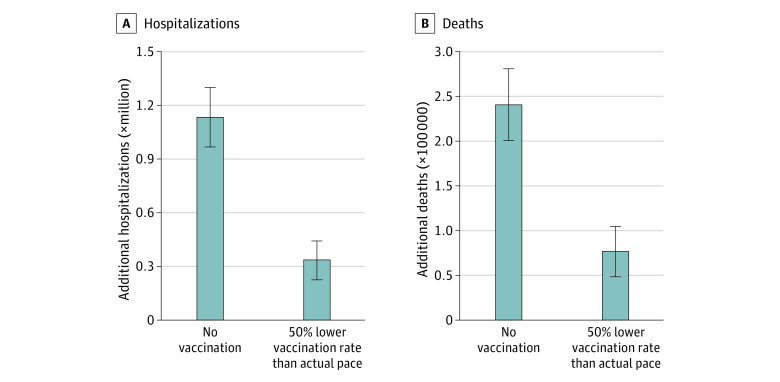
Association of COVID-19 Vaccination With Disease Outcomes Projected additional hospitalizations (A) and deaths (B) that would have occurred under the counterfactual scenarios of no vaccination and 50% reduced daily vaccination rate. Bars indicate the mean of estimates and error bars represent the 95% credible intervals.

Compared with the no vaccination scenario, the actual vaccination campaign saved an estimated 240 797 (95% CrI, 200 665-281 230) lives and prevented an estimated 1 133 617 (95% CrI, 967 487-1 301 881) hospitalizations from December 12, 2020, to June 30, 2021. The number of cases averted during the same period was projected to exceed 14 million. Vaccination prevented a wave of COVID-19 cases driven by the Alpha variant that would have occurred in April 2021 without vaccination (Figure 1), with a projected peak of 4409 (95% CrI, 2865-6312) deaths and 17 979 (95% CrI, 13 191-23 219) hospitalizations. Under the second counterfactual with daily vaccination rates at half the reported pace, we projected that the US would have still endured an additional 77 283 (95% CrI, 48 499-104 519) deaths and 336 000 (95% CrI, 225 330- 440 109) hospitalizations (Figure 2).

## Discussion

Our analytical model suggested that the US COVID-19 vaccination program was associated with a reduction in the total hospitalizations and deaths by nearly half during the first 6 months of 2021. It was also associated with decreased impact of the more transmissible and lethal Alpha variant that was circulating during the same period. As new variants of SARS-CoV-2 continue to emerge, a renewed commitment to vaccine access, particularly among underserved groups and in counties with low vaccination coverage, will be crucial to preventing avoidable COVID-19 cases and bringing the pandemic to a close.

Limitations of our model included the use of reported cases for fitting, which may not reflect the true incidence. This fit does not completely match the temporal trends of reported hospitalizations and deaths. The model was nationally homogeneous; however, parameters may have varied across geographic regions. Furthermore, we did not consider waning immunity after vaccination or recovery within the study time frame.

## Notes

Supplement.
eMethods.
**eTable 1.** Mixing Patterns and the Daily Number of Contacts Derived From Empirical Observations **eTable 2.** Model Parameters Associated With Hospitalization of Severe Cases **eTable 3.** Estimated Vaccine Efficacies (%) and Their 95% CIs From Published Studies
eReferences.
