# Supplementary material for: Estimating COVID-19 Infections, Hospitalizations, and Deaths Following the US Vaccination Campaigns During the Pandemic
Source: JAMA Netw Open. 2022 Jan 11;5(1):e2142725. doi: 10.1001/jamanetworkopen.2021.42725 (PMC8753510; doi:10.1001/jamanetworkopen.2021.42725)
Supplement: Supplement. — eMethods. eTable 1. Mixing Patterns and the Daily Number of Contacts Derived From Empirical Observations eTable 2. Model Parameters Associated With Hospitalization of Severe Cases eTable 3. Estimated Vaccine Efficacies (%) and Their 95% CIs From Published Studies eReferences. [file jamanetwopen-e2142725-s001.pdf]

Supplemental Online Content

Vilches TN, Moghadas SM, Sah P, et al. Estimating COVID-19 infections, hospitalizations, and deaths following the US vaccination campaigns during the pandemic. *JAMA Netw Open*. 2022; 5(1):e2142725. doi:10.1001/jamanetworkopen.2021.42725

eMethods.
eTable 1. Mixing Patterns and the Daily Number of Contacts Derived From Empirical Observations
eTable 2. Model Parameters Associated With Hospitalization of Severe Cases
eTable 3. Estimated Vaccine Efficacies (%) and Their 95% CIs From Published Studies
eReferences.

This supplemental material has been provided by the authors to give readers additional information about their work.

© 2022 Vilches TN et al. *JAMA Network Open.*

eMethods

Model structure

We extended our previous agent-based model of COVID-19 transmission and vaccination [1,2] to include B.1.1.7 (Alpha), P.1 (Gamma) and B.1.617.2 (Delta) variants of SARS-CoV-2 with different transmissibilities in addition to the original pandemic strain. The model implemented the natural history of disease with epidemiological classes for susceptible; exposed and infected (not yet infectious); asymptomatic (and infectious); pre-symptomatic (and infectious); symptomatic (and infectious) with either mild or severe illness; recovered; and dead. The population was stratified into six age groups of 0 to 4, 5 to 19, 20 to 49, 50 to 64, 65 to 79, and 80+ years and incorporated age-specific risk of hospitalizations and deaths, contact patterns, and a two-dose vaccination rollout. Daily contacts between individuals were sampled from a negative-binomial distribution parameterized (eTable 1) using empirical data on pre-pandemic and pandemic-era interactions [3,4].

Transmissibility

Risk of infection for susceptible individuals depended probabilistically on their interaction with infectious individuals in the pre-symptomatic, symptomatic, or asymptomatic stages of infection. The transmission probability of the original strain of SARS-CoV-2 was calibrated by fitting the model to case incidence data per 100,000 population in the entire US from October 1, 2020, to June 30, 2021.[5] We chose October 1 as the starting point for our calibration and simulations because it was a time of a relatively low incidence preceding the fall/winter wave in the US. The calibration (in the presence of only the original strain of SARS-CoV-2) resulted in a transmission probability of 0.109. This transmission probability corresponds to an effective reproduction number of 1.17 in early October 2020 [6], which accounted for the effect of non-pharmaceutical interventions (NPIs) in simulated scenarios. We then introduced the Alpha variant on December 1, 2020 (12 days prior to the start of vaccination in the US) [7] with a 50% higher transmissibility compared to the original strain [8–10]. We introduced the Gamma variant on January 5 [11] and the Delta variant (B.1.617.2) in the model on March 13, 2021 [12]. The transmissibility of Gamma was 60% higher compared to the original strain [13], while the transmissibility of the Delta variant was set as 30% higher than the Alpha variant [14].

Disease dynamics

We parameterized the infectivity of asymptomatic, mild symptomatic, and severe symptomatic individuals to be 26%, 44%, and 89% relative to the pre-symptomatic stage [15–17]. We assumed that these relative infectivities remained the same for all variants in the model. The incubation period was sampled from a log-normal distribution with a mean of 5.2 days [18], and parameters of 1.434 (shape) and 0.661 (scale). An age-dependent proportion of infected individuals progressed to a pre-symptomatic stage with a mean duration of 2.3 days, sampled from a Gamma distribution with parameters of 1.058 (shape) and 2.17 (scale) [16,19]. Pre-symptomatic cases developed symptomatic disease with a mean infectious period of 3.2 days, which was also sampled from a Gamma distribution with parameters of 2.77 (shape) and 1.1563 (scale) [20,21]. The remaining proportion of infected individuals experienced asymptomatic infection until recovery, with a mean infectious period of 5 days sampled from a Gamma distribution with parameters of 5 (shape) and 1 (scale) [20,21].

Recent studies indicate that antibodies from prior infection with other variants of SARS-CoV-2 may have reduced neutralizing activity against Gamma and Delta [22–25]. We therefore assumed that both the Gamma and Delta variants evade naturally acquired immunity by an average of

© 2022 Vilches TN et al. *JAMA Network Open.*

21% (95% CI: 11-36%) [26,27]. This evasion rate was implemented as a reduction of immune protection for individuals recovered from the original strain or the Alpha variant, corresponding to an average transmission probability of 0.21×0.109= 0.0229 per contact. We further assumed that recovery from infection due to the Gamma or Delta variant provides protection against all variants in the model, preventing reinfection for at least one year.

Infection outcomes
We assumed that asymptomatic and mild symptomatic cases recover from infection without hospitalization. A proportion of those with severe disease were hospitalized within 2-5 days of symptom onset [28,29] and were therefore removed from the transmission chain. We also assumed that all symptomatic cases who were not hospitalized self-isolated within 24 hours of symptom onset, and reduced their number of daily contacts by an additional 72% (eTable 1). Intensive care unit (ICU) and non-ICU hospitalization rates were parameterized (eTable 2) by clinical and epidemiological data stratified by age and comorbidities [30–32]. Infection with Alpha variant was associated with 64% higher risk of death [9,10], and infections with Gamma or Delta variants were assigned the case fatality of the original strain.

Vaccination
We implemented a two-dose vaccination campaign with a sequential prioritization of: (i) healthcare workers (5% of the total population) [35], adults with comorbidities, and those aged 65 and older; and (ii) other individuals aged 16-64 [36,37]. Based on vaccine uptake data, we assigned 60% probability of vaccination for individuals aged 40-64 years and 40% vaccination probability for individuals aged 16-39 years [38]. The minimum age-eligibility for vaccination was 16 years before May 13, 2021 after which children aged 12 to 15 years became eligible for vaccination. We used reported daily vaccine doses administered since the start of vaccination to parameterize a rolling 7-day average of vaccine distribution per 100,000 population [39].

We specified Pfizer-BioNTech vaccines with an interval of 21 days between the first and second doses [40]. This interval was 28 days for Moderna vaccines [41]. We parameterized the model with published estimates of vaccine efficacy following each dose of Pfizer-BioNTech and Moderna vaccines against infection, symptomatic disease, and severe disease caused by the original strain [1,42]. The mean efficacies, reported in eTable 3, were implemented in the model as a reduction of transmission probability (for efficacy against infection), reduction in probability of developing symptomatic disease, and reduction of severe illness if symptomatic disease occurred.

Data sources
The overall demographic characteristics and temporal distribution of individuals receiving COVID-19 vaccines was obtained from the CDC data repository [39]. Daily COVID-19 infections (i.e., confirmed by a test), and deaths (reported as associated with COVID-19) were obtained from New York Times Github repository [43]. Daily new COVID-19 hospital admissions were retrieved from the HealthGov data repository [44].

Model implementation
Assuming 10% pre-existing immunity generated by the original strain prior to October 2020 [56,57], we simulated the model with a population of 100,000 individuals from October 1, 2020 to December 1, 2021. To incorporate the age distribution of pre-existing immunity in the population, we ran the model with only the original strain in the absence of vaccination and determined the infection rates in different age groups when the overall attack rate reached 10%. The distribution of this immunity was used to parameterize the initial population at the start of simulations.

© 2022 Vilches TN et al. *JAMA Network Open.*

On April 2, the guidelines by the US Centers for Disease Control and Prevention indicated a minimal risk for fully vaccinated individuals to travel and engage in certain social activities while taking COVID-19 precautions [58]. We therefore allowed vaccinated individuals to return to pre-pandemic behaviour 14 days after the second dose of vaccine from April 3, 2021.

During the calibration process (in the presence of only the original strain of SARS-CoV-2), we used a 50% lower rate of contacts (compared to pre-pandemic normal behaviour) and determined the transmission probability of 0.109 per contact in the pre-symptomatic stage of infection. Transmissibility during other stages of infection was adjusted according to their infectivity relative to the pre-symptomatic stage. The transmission probability obtained during the calibration corresponds to an effective reproduction number of 1.17 in early October 2020 [6], accounting for the effect of NPIs. After the calibration, the transmission probability of the original strain remained fixed and the age-specific contact rates were adjusted throughout the simulations (in all scenarios with and without vaccination) to implicitly account for the change in various NPIs implemented in the US and fit the model to observed incidence data. The model was implemented in Julia, which is an open-source, high-performance, dynamic programming language that allows rapid analysis of computationally intensive problems, such as agent-based modelling. Simulations were averaged over 500 independent Monte-Carlo realizations, and 95% credible intervals derived using a bias-corrected and accelerated bootstrap method. The simulation codes are available at:
https://github.com/thomasvilches/multiple_strains/tree/rapid_vaccination

© 2022 Vilches TN et al. *JAMA Network Open.*

eTable 1. Mixing Patterns and the Daily Number of Contacts Derived From Empirical Observations[a]

Age group	Proportion of contacts between age groups					No. of daily contacts without self-isolation Mean (SD)	No. of daily contacts for self-isolated individuals Mean (SD)
	**0-4**	**5-19**	**20-49**	**50-65**	**65+**		
0-4	0.2287	0.1839	0.4219	0.1116	0.0539	10.21 (7.65)	2.86 (2.14)
5-19	0.0276	0.5964	0.2878	0.0591	0.0291	16.793 (11.7201)	4.70 (3.28)
20-49	0.0376	0.1454	0.6253	0.1423	0.0494	13.795 (10.5045)	3.86 (2.95)
50-65	0.0242	0.1094	0.4867	0.2723	0.1074	11.2669 (9.5935)	3.15 (2.66)
65+	0.0207	0.1083	0.4071	0.2193	0.2446	8.0027 (6.9638)	2.24 (1.95)

[a]Daily numbers of contacts were sampled from negative binomial distributions for different[3,4] scenarios

eTable 2. Model Parameters Associated With Hospitalization of Severe Cases

Proportion of severe cases hospitalized with one or more comorbidities		100%	[30–32]
	Non-ICU	60.4%	
	ICU	39.6%	
Proportion of severe cases hospitalized without any comorbidities		10.8%	[30–32]
	Non-ICU	75%	
	ICU	25%	
Length of non-ICU stay (days)		Gamma(shape: 4.5, scale: 2.75)	Derived from [33,34]
Length of ICU stay (days)		Gamma(shape: 4.5, scale: 2.75) + 2	Derived from [33,34]

© 2022 Vilches TN et al. *JAMA Network Open.*

eTable 3. Estimated Vaccine Efficacies (%) and Their 95% CIs From Published Studies

Vaccine efficacy (%)	Weeks after the first dose		Weeks after the second dose		Reference
Original strain	1-2	3	1-2	>2	41,45–49
Infection	None	46 (40, 51)	60 (53, 66)	86.1 (82.4, 89.1)	
Symptomatic disease	None	57 (50, 63)	66 (57, 73)	94 (87, 98)	
Severe disease	None	62 (39, 80)	80 (59, 94)	92 (75, 100)	
Alpha variant	1-2	3	1-2	>2	50–52
Infection	None	29.5 (22.9, 35.5)	60 (53, 66)	89.5 (85.9, 92.3)	
Symptomatic disease	None	53.6 (50, 63)	62 (57, 73)	93.7 (91.6, 95.3)	
Severe disease	None	54.1 (26.1, 71.9)	80 (59, 94)	94 (87, 98)	
Gamma variant[a]	1-2	3	1-2	>2	50,51
Infection	None	36.8 (32., 40.8)	48 (42.4, 52)	73.6 (70.4, 76)	
Symptomatic disease	None	33.2 (8.3, 51.4)	66 (57, 73)	94 (87, 98)	
Severe disease	None	34 (0, 50)	68 (64, 75)	97.4 (92.2, 99.5)	
Delta variant	1-2	3	1-2	>2	52–55
Infection	None	36.8 (32.0, 40.8)	48 (42.4, 52)	64 (57, 70)	
Symptomatic disease	None	33.5 (20.6, 44.3)	62 (57, 73)	88 (85.3, 90.1)	

© 2022 Vilches TN et al. *JAMA Network Open.*

Severe disease	None	34 (0, 50)	68 (64, 75)	80 (73–85)	

[a] Vaccine efficacy against the Gamma variant was assumed to be the same as those reported for Beta.

eReferences

1. Sah P, Vilches TN, Moghadas SM, et al. Accelerated vaccine rollout is imperative to mitigate highly transmissible COVID-19 variants. *EClinicalMedicine*. 2021;35. doi:10.1016/j.eclinm.2021.100865

2. Moghadas SM, Vilches TN, Zhang K, et al. The impact of vaccination on COVID-19 outbreaks in the United States. *Clin Infect Dis*. Published online January 30, 2021. doi:10.1093/cid/ciab079

3. Mossong J, Hens N, Jit M, et al. Social contacts and mixing patterns relevant to the spread of infectious diseases. *PLoS Med*. 2008;5(3):e74.

4. Jarvis CI, Van Zandvoort K, Gimma A, et al. Quantifying the impact of physical distance measures on the transmission of COVID-19 in the UK. *BMC Med*. 2020;18(1):124.

5. The New York Times. *Coronavirus (Covid-19) Data in the United States*. Github; 2021. Accessed May 1, 2021. https://github.com/nytimes/covid-19-data

6. Krieger M, Owens T. *Rt COVID-19*. Accessed May 2, 2021. https://github.com/rtcovidlive/

7. Galloway SE, Paul P, MacCannell DR, et al. Emergence of SARS-CoV-2 B.1.1.7 Lineage - United States, December 29, 2020-January 12, 2021. *MMWR Morb Mortal Wkly Rep*. 2021;70(3):95-99.

8. Davies NG, Abbott S, Barnard RC, et al. Estimated transmissibility and impact of SARS-CoV-2 lineage B.1.1.7 in England. *Science*. 2021;372(6538). doi:10.1126/science.abg3055

9. Davies NG, Jarvis CI, CMMID COVID-19 Working Group, et al. Increased mortality in community-tested cases of SARS-CoV-2 lineage B.1.1.7. *Nature*. Published online March 15, 2021. doi:10.1038/s41586-021-03426-1

10. Challen R, Brooks-Pollock E, Read JM, Dyson L, Tsaneva-Atanasova K, Danon L. Risk of mortality in patients infected with SARS-CoV-2 variant of concern 202012/1: matched cohort study. *BMJ*. 2021;372:n579.

11. CDC. Science brief: Emerging SARS-CoV-2 variants. Published March 26, 2021. Accessed May 2, 2021. https://www.cdc.gov/coronavirus/2019-ncov/science/science-briefs/scientific-brief-emerging-variants.html

12. Bolze A, Cirulli ET, Luo S, et al. Rapid displacement of SARS-CoV-2 variant B.1.1.7 by B.1.617.2 and P.1 in the United States. *medRxiv*. Published online June 30, 2021:2021.06.20.21259195.

13. Faria NR, Mellan TA, Whittaker C, et al. Genomics and epidemiology of the P.1 SARS-CoV-2 lineage in Manaus, Brazil. *Science*. 2021;372(6544):815-821.

14. Allen AH, Vusirikala A, Flannagan J, et al. Increased household transmission of COVID-19 cases associated with SARS-CoV-2 Variant of Concern B.1.617.2: a national case- control study. Accessed July 1, 2021. https://khub.net/documents/135939561/405676950/Increased+Household+Transmission+o

© 2022 Vilches TN et al. *JAMA Network Open.*

f+COVID-19+Cases+-+national+case+study.pdf/7f7764fb-ecb0-da31-77b3-b1a8ef7be9aa

15. Ferretti L, Wymant C, Kendall M, et al. Quantifying SARS-CoV-2 transmission suggests epidemic control with digital contact tracing. *Science*. 2020;368(6491). doi:10.1126/science.abb6936

16. Moghadas SM, Fitzpatrick MC, Sah P, et al. The implications of silent transmission for the control of COVID-19 outbreaks. *Proc Natl Acad Sci U S A*. 2020;117(30):17513-17515.

17. Sayampanathan AA, Heng CS, Pin PH, Pang J, Leong TY, Lee VJ. Infectivity of asymptomatic versus symptomatic COVID-19. *Lancet*. 2021;397(10269):93-94.

18. Li Q, Guan X, Wu P, et al. Early Transmission Dynamics in Wuhan, China, of Novel Coronavirus-Infected Pneumonia. *N Engl J Med*. 2020;382(13):1199-1207.

19. He X, Lau EHY, Wu P, et al. Temporal dynamics in viral shedding and transmissibility of COVID-19. *Nat Med*. 2020;26(5):672-675.

20. Li R, Pei S, Chen B, et al. Substantial undocumented infection facilitates the rapid dissemination of novel coronavirus (SARS-CoV-2). *Science*. 2020;368(6490):489-493.

21. Gatto M, Bertuzzo E, Mari L, et al. Spread and dynamics of the COVID-19 epidemic in Italy: Effects of emergency containment measures. *Proc Natl Acad Sci U S A*. 2020;117(19):10484-10491.

22. Zhou D, Dejnirattisai W, Supasa P, et al. Evidence of escape of SARS-CoV-2 variant B.1.351 from natural and vaccine-induced sera. *Cell*. 2021;184(9):2348-2361.e6.

23. Planas D, Bruel T, Grzelak L, et al. Sensitivity of infectious SARS-CoV-2 B.1.1.7 and B.1.351 variants to neutralizing antibodies. *Nat Med*. Published online March 26, 2021. doi:10.1038/s41591-021-01318-5

24. Vaidyanathan G. Coronavirus variants are spreading in India - what scientists know so far. *Nature*. 2021;593(7859):321-322.

25. Liu C, Ginn HM, Dejnirattisai W, et al. Reduced neutralization of SARS-CoV-2 B.1.617 by vaccine and convalescent serum. *Cell*. Published online June 17, 2021. doi:10.1016/j.cell.2021.06.020

26. Centre for Mathematical Modelling of Infectious Diseases. Estimates of severity and transmissibility of novel SARS-CoV-2 variant 501Y.V2 in South Africa. Estimates of severity and transmissibility of novel SARS-CoV-2 variant 501Y.V2 in South Africa. Published January 11, 2021. Accessed May 2, 2021. https://cmmid.github.io/topics/covid19/sa-novel-variant.html

27. Jangra S, Ye C, Rathnasinghe R, et al. SARS-CoV-2 spike E484K mutation reduces antibody neutralisation. *Lancet Microbe*. 2021;2(7):e283-e284.

28. Shoukat A, Wells CR, Langley JM, Singer BH, Galvani AP, Moghadas SM. Projecting demand for critical care beds during COVID-19 outbreaks in Canada. *CMAJ*. 2020;192(19):E489-E496.

29. Moghadas SM, Shoukat A, Fitzpatrick MC, et al. Projecting hospital utilization during the

COVID-19 outbreaks in the United States. *Proc Natl Acad Sci U S A*. 2020;117(16):9122-9126.

30. Garg S, Kim L, Whitaker M, et al. Hospitalization rates and characteristics of patients hospitalized with laboratory-confirmed Coronavirus disease 2019 - COVID-NET, 14 states, March 1-30, 2020. *MMWR Morb Mortal Wkly Rep*. 2020;69(15):458-464.

31. Team CC-19 R, CDC COVID-19 Response Team, Chow N, et al. Preliminary Estimates of the Prevalence of Selected Underlying Health Conditions Among Patients with Coronavirus Disease 2019 — United States, February 12–March 28, 2020. *MMWR Morbidity and Mortality Weekly Report*. 2020;69(13):382-386. doi:10.15585/mmwr.mm6913e2

32. Nyberg T, Twohig KA, Harris RJ, et al. Risk of hospital admission for patients with SARS-CoV-2 variant B.1.1.7: cohort analysis. *BMJ*. 2021;373:n1412.

33. Guan W-J, Ni Z-Y, Hu Y, et al. Clinical Characteristics of Coronavirus Disease 2019 in China. *N Engl J Med*. 2020;382(18):1708-1720.

34. Sanche S, Lin YT, Xu C, Romero-Severson E, Hengartner N, Ke R. High Contagiousness and Rapid Spread of Severe Acute Respiratory Syndrome Coronavirus 2. *Emerg Infect Dis*. 2020;26(7):1470-1477.

35. U.S. Bureau of Labor Statistics. Number of hospitals and hospital employment in each state in 2019. Published April 6, 2020. Accessed May 2, 2021. https://www.bls.gov/opub/ted/2020/number-of-hospitals-and-hospital-employment-in-each-state-in-2019.htm

36. Committee on Equitable Allocation of Vaccine for the Novel Coronavirus, National Academy of Medicine, National Academies of Sciences, Engineering, and Medicine. *Discussion Draft of the Preliminary Framework for Equitable Allocation of COVID-19 Vaccine*. National Academies Press; 2020.

37. CDC. How CDC is making COVID-19 vaccine recommendations. Published April 29, 2021. Accessed May 2, 2021. https://www.cdc.gov/coronavirus/2019-ncov/vaccines/recommendations-process.html?CDC_AA_refVal=https%3A%2F%2Fwww.cdc.gov%2Fcoronavirus%2F2019-ncov%2Fvaccines%2Frecommendations.html

38. CDC. COVID Data Tracker. Published March 28, 2020. Accessed May 18, 2021. https://covid.cdc.gov/covid-data-tracker/

39. IISInfo. COVID-19 vaccination demographics in the United States,national. Published online May 24, 2021. Accessed November 2, 2021. https://data.cdc.gov/Vaccinations/COVID-19-Vaccination-Demographics-in-the-United-St/km4m-vcsb

40. Anderson EJ, Rouphael NG, Widge AT, et al. Safety and Immunogenicity of SARS-CoV-2 mRNA-1273 Vaccine in Older Adults. *N Engl J Med*. 2020;383(25):2427-2438.

41. Polack FP, Thomas SJ, Kitchin N, et al. Safety and Efficacy of the BNT162b2 mRNA Covid-19 Vaccine. *N Engl J Med*. 2020;383(27):2603-2615.

© 2022 Vilches TN et al. *JAMA Network Open.*

42. Moghadas SM, Vilches TN, Zhang K, et al. Evaluation of COVID-19 vaccination strategies with a delayed second dose. *PLoS Biol*. 2021;19(4):e3001211.

43. *Covid-19-Data: An Ongoing Repository of Data on Coronavirus Cases and Deaths in the U.S*. Github Accessed November 2, 2021. https://github.com/nytimes/covid-19-data

44. U.S. Department of Health & Human Services. COVID-19 Reported Patient Impact and Hospital Capacity by State Timeseries. Published online December 14, 2020. Accessed November 2, 2021. https://healthdata.gov/Hospital/COVID-19-Reported-Patient-Impact-and-Hospital-Capa/g62h-syeh

45. Dagan N, Barda N, Kepten E, et al. BNT162b2 mRNA Covid-19 Vaccine in a Nationwide Mass Vaccination Setting. *N Engl J Med*. 2021;384(15):1412-1423.

46. U.S. Food and Drug Administration. *Vaccines and Related Biological Products Advisory Committee December 10, 2020 Meeting Briefing Document*. U.S. Food and Drug Administration; 2020. Accessed July 2, 2021. https://www.fda.gov/media/144246/

47. Lipsitch M, Kahn R. Interpreting vaccine efficacy trial results for infection and transmission. *Vaccine*. Published online June 12, 2021. doi:10.1016/j.vaccine.2021.06.011

48. Chodick G, Tene L, Patalon T, et al. Assessment of Effectiveness of 1 Dose of BNT162b2 Vaccine for SARS-CoV-2 Infection 13 to 24 Days After Immunization. *JAMA Netw Open*. 2021;4(6):e2115985.

49. Pawlowski C, Lenehan P, Puranik A, et al. FDA-authorized mRNA COVID-19 vaccines are effective per real-world evidence synthesized across a multi-state health system. *Med (N Y)*. Published online June 29, 2021. doi:10.1016/j.medj.2021.06.007

50. Abu-Raddad LJ, Chemaitelly H, Butt AA, National Study Group for COVID-19 Vaccination. Effectiveness of the BNT162b2 Covid-19 Vaccine against the B.1.1.7 and B.1.351 Variants. *N Engl J Med*. Published online May 5, 2021. doi:10.1056/NEJMc2104974

51. Vizient, Inc. *COVID-19 Vaccine Candidates*.; 2021. https://www.vizientinc.com/-/media/documents/sitecorepublishingdocuments/public/covid19_sidebyside_vaccinecompare.pdf

52. Lopez Bernal J, Andrews N, Gower C, et al. Effectiveness of Covid-19 Vaccines against the B.1.617.2 (Delta) Variant. *N Engl J Med*. 2021;385(7):585-594.

53. Pouwels KB, Pritchard E, Matthews P, et al. Impact of Delta on viral burden and vaccine effectiveness against new SARS-CoV-2 infections in the UK. *bioRxiv*. Published online August 24, 2021. doi:10.1101/2021.08.18.21262237

54. Bernal JL, Andrews N, Gower C, et al. Effectiveness of COVID-19 vaccines against the B.1.617.2 variant. *bioRxiv*. Published online May 24, 2021. doi:10.1101/2021.05.22.21257658

55. Grannis SJ, Rowley EA, Ong TC, et al. Interim Estimates of COVID-19 Vaccine Effectiveness Against COVID-19-Associated Emergency Department or Urgent Care Clinic Encounters and Hospitalizations Among Adults During SARS-CoV-2 B.1.617.2 (Delta) Variant Predominance - Nine States, June-August 2021. *MMWR Morb Mortal Wkly Rep*.

© 2022 Vilches TN et al. *JAMA Network Open.*

2021;70(37):1291-1293.

56. Bajema KL, Wiegand RE, Cuffe K, et al. Estimated SARS-CoV-2 Seroprevalence in the US as of September 2020. *JAMA Intern Med*. 2021;181(4):450-460.

57. Arora R, Yan T. SeroTracker. SeroTracker. Published May 2, 2021. Accessed May 2, 2021. https://serotracker.com

58. CDC. Interim public health recommendations for fully vaccinated people. Published April 30, 2021. Accessed May 2, 2021. https://www.cdc.gov/coronavirus/2019-ncov/vaccines/fully-vaccinated-guidance.html

© 2022 Vilches TN et al. *JAMA Network Open.*
